# Supplementary material for: Choosing an analytic approach: key study design considerations in state policy evaluation
Source: Health Serv Outcomes Res Methodol. 2025 Nov 17;26(1):3–25. doi: 10.1007/s10742-025-00369-2 (PMC12967469; doi:10.1007/s10742-025-00369-2)
Supplement: Supplementary file 2 — Supplementary Material 2 [file 10742_2025_369_MOESM2_ESM.docx]

# Manuscript Code Appendix

# Choosing an analytic approach: Key study design considerations in state policy evaluation

# Elizabeth M. Stone, Megan S. Schuler, Elizabeth A. Stuart, Max Rubinstein, Max Griswold, Bradley D. Stein, Beth Ann Griffin

# Code to use for reading in the data set

# setwd("FILL IN FOLDER LOCATION WHERE SAVED CASE STUDY DATASET")

casestudy_data<-read.table("casestudy_data.csv",sep=",",h=T)

# Uses casestudy_data

dta <- casestudy_data

# Installation notes for new packages:

# When its the first time using many of these libraries:

# Use install.packages("libraryname")

# For augsynth - you will need to type:

# install.packages("devtools")

# devtools::install_github("ebenmichael/augsynth")

################################################################################

################################################################################

# 1. Interrupted time series (ITS)

# Include only 2014 cohort

dta1 <- dta[dta$standing_order_cohort == 2014 ,]

# Generate variable of time since policy implementation (2014)

dta1$time_since_trt <- ifelse(dta1$year > 2014, dta1$year - 2014, 0)

ts <- lm(OD_rate ~ year + standing_order_policy + time_since_trt + as.factor(state_id) + unemployment_rate, data=dta1)

# Perform needed cluster adjustment to standard errors

library(lmtest)

library(sandwich)

coeftest(ts, vcovCL(x = ts, cluster = dta7$state, type = "HC1"))

# Code needed for graphic

tsc <- coef(ts)

# Observed data for untreated units

plot(dta1$year,

dta1$OD_rate, bty = 'n', xaxt='n',

xlab = "Year", ylab = "OD rate",

ylim = c(0, 40), xlim = c(2000, 2019),

col = rgb(0.5, 0.5, 0.5, 0.5), pch = 19)

axis(side = 1, at = seq(2000, 2018, 2))

# Post-treatment year

abline(v = 2014, col = rgb(1, 0.5, 0, 0.8), lw = 2, lty = 2)

# E[Y*|X = 0 & year < post]

segments(y0 = tsc[["(Intercept)"]] + 2000*tsc[["year"]] + mean(dta1$unemployment_rate)*tsc[["unemployment_rate"]],

y1 = tsc[["(Intercept)"]] + 2014*tsc[["year"]] + mean(dta1$unemployment_rate)*tsc[["unemployment_rate"]],

x0 = 2000, x1 = 2014,

col = rgb(0.65, 0.7, 0.95), lwd = 3)

# E[Y* = 0 & year > post]

segments(y0 = tsc[["(Intercept)"]] + 2014*tsc[["year"]] + mean(dta1$unemployment_rate)*tsc[["unemployment_rate"]],

y1 = tsc[["(Intercept)"]] + 2017*tsc[["year"]] + mean(dta1$unemployment_rate)*tsc[["unemployment_rate"]],

x0 = 2014, x1 = 2017,

col = rgb(0.65, 0.7, 0.95), lwd = 3, lty = 2)

# E[Y*|X = 1 & year > post]

segments(y0 = tsc[["(Intercept)"]] + 2014*tsc[["year"]] + tsc[["standing_order_policy"]] + mean(dta1$unemployment_rate)*tsc[["unemployment_rate"]],

y1 = tsc[["(Intercept)"]] + 2017*tsc[["year"]]+ tsc[["standing_order_policy"]] + 3*tsc[["time_since_trt"]] + mean(dta1$unemployment_rate)*tsc[["unemployment_rate"]],

x0 = 2014, x1 = 2017,

col = rgb(0.65, 0.7, 0.95), lwd = 3)

################################################################################

################################################################################

# 2: ITS with multiple baselines

# Create separate datasets for 2014, 2015, & 2016 cohorts

dta2_2014 <- dta[dta$standing_order_cohort == 2014 ,]

dta2_2015 <- dta[dta$standing_order_cohort == 2015 ,]

dta2_2016 <- dta[dta$standing_order_cohort == 2016 ,]

# Generate variable of time since policy implementation

dta2_2014$time_since_trt <- ifelse(dta2_2014$year > 2014, dta2_2014$year - 2014, 0)

dta2_2015$time_since_trt <- ifelse(dta2_2015$year > 2015, dta2_2015$year - 2015, 0)

dta2_2016$time_since_trt <- ifelse(dta2_2016$year > 2016, dta2_2016$year - 2016, 0)

# Time series regression for each cohort

ts_2014 <- lm(OD_rate ~ year + standing_order_policy + time_since_trt + unemployment_rate, data=dta2_2014)

ts_2015 <- lm(OD_rate ~ year + standing_order_policy + time_since_trt + unemployment_rate, data=dta2_2015)

ts_2016 <- lm(OD_rate ~ year + standing_order_policy + time_since_trt + unemployment_rate, data=dta2_2016)

# Perform needed cluster adjustment to standard errors

library(lmtest)

library(sandwich)

output_2014 <- coeftest(ts_2014, vcovCL(x = ts_2014, cluster = dta2_2014$state, type = "HC1"))

output_2015 <- coeftest(ts_2015, vcovCL(x = ts_2015, cluster = dta2_2015$state, type = "HC1"))

output_2016 <- coeftest(ts_2016, vcovCL(x = ts_2016, cluster = dta2_2016$state, type = "HC1"))

# Function to extract coefficient and p-value for standing_order_policy & time_since_trt

extract_coef <- function(coeftest_output) {

sop_coef_value <- coeftest_output["standing_order_policy", "Estimate"]

sop_p_value <- coeftest_output["standing_order_policy", "Pr(>|t|)"]

tst_coef_value <- coeftest_output["time_since_trt", "Estimate"]

tst_p_value <- coeftest_output["time_since_trt", "Pr(>|t|)"]

return(c(sop_coef_value, sop_p_value, tst_coef_value, tst_p_value))

}

results2014 <- extract_coef(output_2014)

results2015 <- extract_coef(output_2015)

results2016 <- extract_coef(output_2016)

# Create a table with model names, coefficients, and p-values

model_names <- c("2014 cohort", "2015 cohort", "2016 cohort")

sop_coef_values <- c(results2014[1], results2015[1], results2016[1])

sop_p_values <- c(results2014[2], results2015[2], results2016[2])

tst_coef_values <- c(results2014[3], results2015[3], results2016[3])

tst_p_values <- c(results2014[4], results2015[4], results2016[4])

model_summary <- data.frame(

Model = model_names,

standing_order_policy = sop_coef_values,

p_value = sop_p_values,

time_since_treatment = tst_coef_values,

p_value = tst_p_values

)

# Print the table

print(model_summary)

################################################################################

################################################################################

# 3: Synthetic control method

# Include only Illinois (2010) and never treated control states

dta3 <- dta[dta$standing_order_cohort == 0 | dta$standing_order_cohort == 2010, ]

library(Synth)

# Create matrices from data that provides inputs for synth

dataprep.out <-

dataprep(foo = dta3,

predictors = "unemployment_rate" ,

predictors.op = "mean" ,

special.predictors = list(

list("OD_rate" , 1999:2009 , "mean")

),

dependent = "OD_rate",

unit.variable = "state_id",

time.variable = "year",

treatment.identifier = 14,

controls.identifier = c(7,13,17,28,37,50,51),

time.predictors.prior = 1999:2009,

time.optimize.ssr = 1999:2009,

time.plot = 1999:2017

)

# Identify weights that create the best synthetic control

synth.out <-synth(dataprep.out)

# Get results tables

print(synth.tables <- synth.tab(

dataprep.res = dataprep.out,

synth.res = synth.out)

)

# Synthetic control plot

path.plot(synth.res = synth.out,

dataprep.res = dataprep.out,

Ylab = c("OD rate"),

Xlab = c("Year"),

Legend = c("Illinois","Synthetic Illinois"),

Legend.position = c("topleft")

)

abline(v = 2010,

lty = 2)

################################################################################

################################################################################

# 4: Augmented synthetic control method

# Include only Illinois (2010) and never treated control states

dta4 <- dta[dta$standing_order_cohort == 0 | dta$standing_order_cohort == 2010, ]

library(magrittr)

library(dplyr)

library(augsynth)

# Augsynth analysis

syn <- augsynth(

OD_rate ~ standing_order_policy | unemployment_rate,

unit = state_id,

time = year,

data = dta4,

progfunc = "ridge",

scm = TRUE,

fixedeff = TRUE

)

# Effect estimates: year-specific ATTs

summary(syn)

# Plot time-specific ATTs with 95% confidence interval

plot(syn)

################################################################################

################################################################################

# 5: Classic difference-in-differences / two-way fixed effects

# Include only 2014 cohort and never treated group

dta5 <- dta[dta$standing_order_cohort == 0 | dta$standing_order_cohort == 2014, ]

# Generate indicator for treatment states (1) vs. control states (0)

dta5$treated_state<-0

dta5$treated_state[dta5$standing_order_cohort > 0] <- 1

# Generate indicator for pre (0) vs. post (1) policy implementation

dta5$standing_order_policy[dta5$year < 2014] <- 0

dta5$standing_order_policy[dta5$year > 2013] <- 1

# Generate interaction term for treatment state X pre/post

dta5$treated_stateXpolicy <- dta5$treated_state * dta5$standing_order_policy

# Difference-in-differences regression model

twfe <- lm(OD_rate ~ treated_stateXpolicy + as.factor(year) + as.factor(state_id) + unemployment_rate, data=dta5)

summary(twfe)

# Perform needed cluster adjustment to standard errors

library(lmtest)

library(sandwich)

coeftest(twfe, vcovCL(x = twfe, cluster = dta5$state, type = "HC1"))

################################################################################

################################################################################

# 6: Event-study / dynamic difference-in-differences

# Include only 2014 cohort and never treated group

dta6 <- dta[dta$standing_order_cohort == 0 | dta$standing_order_cohort == 2014, ]

# Generate indicator for treatment states (1) vs. control states (0)

dta6$treated_state<-0

dta6$treated_state[dta6$standing_order_cohort > 0] <- 1

# Generate time categorical variable with reference period of the time before treatment

dta6$time_since_trt <-0

dta6$time_since_trt[dta6$year == 1999] <- -14

dta6$time_since_trt[dta6$year == 2000] <- -13

dta6$time_since_trt[dta6$year == 2001] <- -12

dta6$time_since_trt[dta6$year == 2002] <- -11

dta6$time_since_trt[dta6$year == 2003] <- -10

dta6$time_since_trt[dta6$year == 2004] <- -9

dta6$time_since_trt[dta6$year == 2005] <- -8

dta6$time_since_trt[dta6$year == 2006] <- -7

dta6$time_since_trt[dta6$year == 2007] <- -6

dta6$time_since_trt[dta6$year == 2008] <- -5

dta6$time_since_trt[dta6$year == 2009] <- -4

dta6$time_since_trt[dta6$year == 2010] <- -3

dta6$time_since_trt[dta6$year == 2011] <- -2

dta6$time_since_trt[dta6$year == 2012] <- -1

dta6$time_since_trt[dta6$year == 2014] <- 1

dta6$time_since_trt[dta6$year == 2015] <- 2

dta6$time_since_trt[dta6$year == 2016] <- 3

dta6$time_since_trt[dta6$year == 2017] <- 4

# Generate interaction terms for treatment state X time categorical variable

dta6$treated_stateXtime <- dta6$treated_state * dta6$time_since_trt

dta6$treated_stateXtime <- as.factor(dta6$treated_stateXtime)

dta6$treated_stateXtime <- relevel (dta6$treated_stateXtime, ref="-1")

# Difference-in-differences regression model

es <- lm(OD_rate ~ as.factor(treated_stateXtime) + as.factor(year) + as.factor(state_id) + unemployment_rate, data=dta6)

summary(es)

# Perform needed cluster adjustment to standard errors

library(lmtest)

library(sandwich)

coeftest(es, vcovCL(x = es, cluster = dta6$state, type = "HC1"))

################################################################################

################################################################################

# 7: Comparative Interrupted Time Series

# Include only 2014 cohort and never treated group

dta7 <- dta[dta$standing_order_cohort == 0 | dta$standing_order_cohort == 2014, ]

# Generate variable of time since policy implementation (2014)

dta7$time_since_trt <-0

dta7$time_since_trt[dta7$year == 2015] <- 1

dta7$time_since_trt[dta7$year == 2016] <- 2

dta7$time_since_trt[dta7$year == 2017] <- 3

# Generate indicator for treatment states (1) vs. control states (0)

dta7$treated_state<-0

dta7$treated_state[dta7$standing_order_cohort > 0] <- 1

dta7$postperiod <- 0

dta7$postperiod[dta7$year >= 2014] <- 1

cts <- lm(OD_rate ~ treated_state*year + treated_state*postperiod + treated_state*time_since_trt + as.factor(state) + unemployment_rate, data = dta7)

# Perform needed cluster adjustment to standard errors

library(lmtest)

library(sandwich)

coeftest(cts, vcovCL(x = cts, cluster = dta7$state, type = "HC1"))

# Use model coefficients to plot trends in treated and control groups:

#alternative graphic

ctsc <- coef(cts)

# Control group:

# E[Y*|X = 0 & year < post]

e_x0_t0 <- c(ctsc[["(Intercept)"]] + 2000*ctsc[["year"]] + mean(dta7$unemployment_rate)*tsc[["unemployment_rate"]],

ctsc[["(Intercept)"]] + 2014*ctsc[["year"]] + mean(dta7$unemployment_rate)*tsc[["unemployment_rate"]])

# E[Y*|X = 0 & year >= post]

e_x0_t1 <- c(ctsc[["(Intercept)"]] + 2014*ctsc[["year"]] + ctsc[['postperiod']] + mean(dta7$unemployment_rate)*tsc[["unemployment_rate"]],

ctsc[["(Intercept)"]] + 2017*ctsc[["year"]] + 3*ctsc[['time_since_trt']] + ctsc[['postperiod']] + mean(dta7$unemployment_rate)*tsc[["unemployment_rate"]])

# Treated group:

# E[Y*|X = 1 & year < post]

e_x1_t0 <- c(ctsc[["(Intercept)"]] + 2000*ctsc[["year"]] + ctsc[['treated_state']] + 2000*ctsc[['treated_state:year']] + mean(dta7$unemployment_rate)*tsc[["unemployment_rate"]],

ctsc[["(Intercept)"]] + 2014*ctsc[["year"]] + ctsc[['treated_state']] + 2014*ctsc[['treated_state:year']] + mean(dta7$unemployment_rate)*tsc[["unemployment_rate"]])

# E[Y*|X = 1 & year >= post]

e_x1_t1 <- c(ctsc[["(Intercept)"]] + 2014*ctsc[["year"]] + ctsc[['treated_state']] + 2014*ctsc[['treated_state:year']] +

+ ctsc[['postperiod']] + ctsc[['treated_state:postperiod']] + mean(dta7$unemployment_rate)*tsc[["unemployment_rate"]],

ctsc[["(Intercept)"]] + 2017*ctsc[["year"]] + ctsc[['treated_state']] + 2018*ctsc[['treated_state:year']] +

3*ctsc[['time_since_trt']] + ctsc[['postperiod']] + ctsc[['treated_state:postperiod']] + 3*ctsc[['treated_state:time_since_trt']] + mean(dta7$unemployment_rate)*tsc[["unemployment_rate"]])

# Counterfactual in treated group:

e_x1_t1_c <- c(ctsc[["(Intercept)"]] + 2014*ctsc[["year"]] + ctsc[['treated_state']] + 2014*ctsc[['treated_state:year']] +

+ ctsc[['postperiod']] + mean(dta7$unemployment_rate)*tsc[["unemployment_rate"]],

ctsc[["(Intercept)"]] + 2017*ctsc[["year"]] + ctsc[['treated_state']] + 2017*ctsc[['treated_state:year']] +

3*ctsc[['time_since_trt']] + ctsc[['postperiod']] + mean(dta7$unemployment_rate)*tsc[["unemployment_rate"]])

# ATT_3 = E[Y*|X = 1 & post == 3] - E[Y*|X = 0 & year == 3]

att <- c(ctsc[["(Intercept)"]] + 2017*ctsc[["year"]] + ctsc[['treated_state']] + 2017*ctsc[['treated_state:year']] +

3*ctsc[['time_since_trt']] + ctsc[['postperiod']] + mean(dta7$unemployment_rate)*tsc[["unemployment_rate"]],

ctsc[["(Intercept)"]] + 2017*ctsc[["year"]] + ctsc[['treated_state']] + 2017*ctsc[['treated_state:year']] +

3*ctsc[['time_since_trt']] + ctsc[['postperiod']] + ctsc[['treated_state:postperiod']] + 3*ctsc[['treated_state:time_since_trt']] + mean(dta7$unemployment_rate)*tsc[["unemployment_rate"]])

# Observed data for untreated units

plot(dta7$year,

dta7$OD_rate, bty = 'n', xaxt='n',

xlab = "Year", ylab = "OD rate",

ylim = c(0, 40), xlim = c(2000, 2019),

col = rgb(0.5, 0.5, 0.5, 0.5), pch = 19)

axis(side = 1, at = seq(2000, 2018, 2))

# Post-treatment year

abline(v = 2014, col = rgb(1, 0.5, 0, 0.8), lw = 2, lty = 2)

# E[Y*|X = 0 & year < post]

segments(y0 = e_x0_t0[1],

y1 = e_x0_t0[2],

x0 = 2000, x1 = 2014,

col = rgb(0.95, 0.7, 0.65), lwd = 3)

# E[Y*|X = 0 & year >= post]

segments(y0 = e_x0_t1[1],

y1 = e_x0_t1[2],

x0 = 2014, x1 = 2017,

col = rgb(0.95, 0.7, 0.65), lwd = 3)

# E[Y*|X = 1 & year < post]

segments(y0 = e_x1_t0[1],

y1 = e_x1_t0[2],

x0 = 2000, x1 = 2014,

col = rgb(0.65, 0.7, 0.95), lwd = 3)

# E[Y*|X = 1 & year >= post]

segments(y0 = e_x1_t1[1],

y1 = e_x1_t1[2],

x0 = 2014, x1 = 2017,

col = rgb(0.65, 0.7, 0.95), lwd = 3)

# Treatment counterfactual

segments(y0 = e_x1_t1_c[1],

y1 = e_x1_t1_c[2],

x0 = 2014, x1 = 2017,

col = rgb(0.65, 0.7, 0.95), lwd = 3, lty = 2)

################################################################################

################################################################################

# 8: Cohort-based DID (Callaway & Sant'Anna staggered difference-in-differences)

# Excludes 2010 (Illinois) and 2013 (North Carolina) cohorts because of small cohort size

dta8 <- subset(dta, !(state %in% c("Illinois", "North Carolina")))

# First, here is the code needed to ensure the policy variable is coded correctly for CSA

# The policy indicator has to be coded as 0 for control states & the year of adoption for treated states

# Pull off implementation dates

dta8$implementation_date <- as.Date(dta8$date_nal_protocol_standing)

# Extract the year and month from the implementation date variable

dta8$cohort_year <- as.numeric(format(dta8$implementation_date, "%Y"))

dta8$cohort_month <- as.numeric(format(dta8$implementation_date, "%m"))

# If the cohort-year is missing, set year to zero so that CSA works correctly

dta8[is.na(dta8$cohort_year),]$cohort_year <- 0

dta8[is.na(dta8$cohort_month),]$cohort_month <- 0

# For treated units, set the cohort year to the next year, if treatment began occurring after June

dta8[dta8$cohort_month > 6,]$cohort_year <- dta8[dta8$cohort_month > 6,]$cohort_year + 1

# If the implementation date is greater than the observed date in the dataset, set

# cohort year to zero (since for the purposes of this dataset, the unit was untreated

# during the study period)

dta8[dta8$cohort_year > max(dta8$year),]$cohort_year <- 0

# Now, check that the new cohort_year variable equals the previous

# "standing_order_cohort" variable which was already available to us

dta8[(dta8$standing_order_cohort != dta8$cohort_year)]

# No rows were printed indicating we have agreement with the existing cohort-year coding!

library(did)

# Estimate group-time average effects

csa_attgt <- att_gt(

yname = "OD_rate",

tname = "year",

idname = "state_id", #important: this idname has to be numeric not a character

gname = "standing_order_cohort", #important: the policy indicator for CSA has to be coded as 0 for control states & the year of adoption for treated states

xformla = ~unemployment_rate,

data = dta8,

allow_unbalanced_panel = TRUE,

control_group = c("nevertreated"),

est_method = "dr")

# Display and graph cohort specific treatment effects

summary(csa_attgt)

ggdid(csa_attgt)

# Average effects by length of exposure to treatment

csa_agg_adj_balance <- aggte(csa_attgt, type = "dynamic")

summary(csa_agg_adj_balance)

ggdid(csa_agg_adj_balance)

# Average effects by length of exposure to treatment with balanced panel (same number of pre- and post-periods from each cohort)

csa_agg_adj_balance <- aggte(csa_attgt, type = "dynamic", min_e = -5, balance_e=0)

summary(csa_agg_adj_balance)

ggdid(csa_agg_adj_balance)

################################################################################

################################################################################

# 9: Imputation-based DID

# Include full sample

library(didimputation)

# Static DID

static <- did_imputation(data = dta, yname = "OD_rate", gname = "standing_order_cohort",

first_stage= ~ unemployment_rate | state + year,

tname = "year", idname = "state")

static

#Event study DID

es <- did_imputation(

data = dta, yname = "OD_rate", gname = "standing_order_cohort",

first_stage= ~ unemployment_rate | state + year,

tname = "year", idname = "state",

# event-study

horizon = TRUE, pretrends = -5:-1

)

es

################################################################################

################################################################################

# 10: Debiased auto-regressive model

# Include full sample

library(data.table)

library(fixest)

library(sandwich)

library(multcomp)

dta10 <- dta

# Create model specification.

mod <- function(df, lag_num, date_name, cov_names = NULL, linear_mod = F){

df <- setDT(df)

if (linear_mod == T){

y_t <- sprintf("y_t%s", 0:(lag_num - 1))

a_t <- sprintf("a_t%s", 1:(lag_num))

form <- paste0(y_t[1], sprintf(" ~ factor(%s) + ", date_name),

paste0(y_t[2:lag_num], collapse = " + "), " + ",

paste0(a_t, collapse = " + "))

if (!is.null(cov_names)){

form <- paste0(form, " + ", paste0(cov_names, collapse = " + "))

}

}else{

# Fixed effects

# First date is the reference category.

dates <- unique(df[, get(date_name)])[-1]

fe_year <- sprintf("alpha_%s", 1:length(dates))

fe_year <- paste0(paste0(fe_year, "*(year == ", dates, ")"), collapse = " + ")

# Outcome lags

y_t <- sprintf("y_t%s", 0:(lag_num - 1))

# Delta terms

delta <- sprintf("delta_%s", 1:(lag_num - 1))

# Time-varying treatment terms

theta_t <- sprintf("theta_t%s", 1:lag_num)

a_t <- sprintf("a_t%s", 0:(lag_num + lag_num - 2))

# Add deltas to specification

delta_terms <- c()

for (i in 1:length(delta)){

delta_terms[i] <- paste0(delta[i], "*(", y_t[i + 1], " - ",

paste0(theta_t, "*", a_t[(i + 1):(i + lag_num)],

collapse = " - "), ")")

}

delta_terms <- paste0(delta_terms, collapse = " + ")

theta_terms <- paste0(theta_t, "*", a_t[1:lag_num], collapse = " + ")

form <- paste0(y_t[1], " ~ beta_0 + ", fe_year, " + ", delta_terms, " + ", theta_terms)

# Add betas for covariates to specification

if (!is.null(cov_names)){

beta_terms <- sprintf("beta_%s", 1:length(cov_names))

beta_terms <- paste0(paste0(beta_terms, "*", cov_names), collapse = " + ")

form <- paste0(form, " + ", beta_terms)

}

}

return(form)

}

# Estimate debiased AR model.

autoreg_debiased <- function(data, lags, outcome_name,

unit_name, date_name, cov_names = NULL){

df <- setDT(data)

treat_lag <- paste0("a_t", 0:(lags + lags - 2))

df[, (treat_lag) := shift(standing_order_policy, n = 0:(lags + lags - 2)), by = unit_name]

outcome_lag <- paste0("y_t", 0:lags)

df[, (outcome_lag) := shift(get(outcome_name), n = 0:lags), by = unit_name]

df[, (date_name) := as.factor(get(date_name))]

# Restrict dataset to the minimum set of rows we can use to estimate the

# model, given the number of lags:

max_lag <- treat_lag[length(treat_lag)]

avail_date <- unique(df[!is.na(get(max_lag)), get(date_name)])

df <- df[get(date_name) %in% avail_date,]

df[, (date_name) := factor(get(date_name), levels = unique(get(date_name)))]

# Get initial starting conditions using a linear model

init_values <- coef(lm(mod(df, lags, linear_mod = T,

date_name = date_name,

cov_names = cov_names), data = df))

# Subtracting due to omitted variables

term_names <- c("beta_0",

sprintf("alpha_%s", 1:(nlevels(df[, get(date_name)]) - 1)),

sprintf("delta_%s", 1:(lags - 1)),

sprintf("theta_t%s", 1:lags))

if (!is.null(cov_names)){

term_names <- c(term_names, sprintf("beta_%s", 1:length(cov_names)))

}

names(init_values) <- term_names

# Using initial try to fit model over 200

# iterations, with smallish steps.

ar_model <- mod(df, lags, date_name = date_name,

cov_names = cov_names)

try_nls <- function(mod, df){

res <- tryCatch(

{

nls(mod,

start = init_values,

data = df,

control = nls.control(minFactor = 1e-10, maxiter = 200))

},

error = function(e){

return(e)

}

)

return(res)

}

mod_fit <- try_nls(ar_model, df)

return(mod_fit)

}

# Run model and use estimated parameters to calculate time-varying effects.

# Specify number of lags

l <- 5

m <- autoreg_debiased(dta10, lags = l, outcome_name = "OD_rate",

unit_name = "state_id", date_name = "year",

cov_names = "unemployment_rate")

mod_fit <- summary(m)

eff_interest <- paste0("theta_t", 1:l)

thetas <- coef(mod_fit)[eff_interest, "Estimate"]

# Overall effects:

theta_sum <- paste0("theta_t", 1:l, collapse = "+")

lincom <- paste0(theta_sum, "=0")

summary(glht(m, lincom))

# Convert to time-varying effects:

estimated_effects <- c()

for (i in 1:length(thetas)){

estimated_effects[i] <- sum(thetas[1:i])

}

# Get variance-covariance matrix, then calculate joint se (standard errors not clustered).

# Simply calculating se of summed RV.

# e.g., b1 + b2 = sqrt(var[b1] + var[b2] + 2Cov[b1, b2])

estimated_se <- c()

vcov_mat <- vcov(m)

vcov_reduced <- which(rownames(vcov_mat) %in% eff_interest)

vcov_mat <- vcov_mat[vcov_reduced, vcov_reduced, drop = F]

sum_se <- function(i, vcov_mat){

vect <- c(rep(1, i), rep(0, ncol(vcov_mat) - i))

se <- sqrt(vect %*% vcov_mat %*% vect)

return(se)

}

for (i in 1:length(thetas)){

estimated_se[i] <- sum_se(i, vcov_mat)

}

print(estimated_effects)

print(estimated_se)

################################################################################

################################################################################

# 11: ASCM extension for multiple units

# Include full sample

library(magrittr)

library(dplyr)

library(augsynth)

# Multisynth analysis

multi_syn <- multisynth(

OD_rate ~ standing_order_policy | unemployment_rate, #policy variable has to be 0/1

unit = state_id, #this idname has to be numeric not a character

time = year,

data = dta,

n_leads=8,

time_cohort = TRUE)

# Effect estimates: average effect by length of exposure to treatment

summary(multi_syn)

# Plot dynamic ATTs (based on length of exposure to treatment) by treatment cohort

plot(multi_syn)

# Plot dynamic ATTs (based on length of exposure to treatment) by treatment cohort

plot(multi_syn, levels = "Average")
